# Alcohol and Liver Function in Women

**DOI:** 10.35946/arcr.v40.2.10

**Published:** 2020-07-02

**Authors:** Haripriya Maddur, Vijay H. Shah

**Affiliations:** 1 Northwestern Feinberg School of Medicine, Northwestern University, Chicago, Illinois; 2 Mayo Clinic, Rochester, Minnesota

**Keywords:** alcohol, estrogen, liver disease, women

## Abstract

Alcohol-related liver disease generally has been ascribed to men because men reportedly consume alcohol at an increased rate and quantity as compared to women. Recent literature has reported, however, that rates of liver disease attributed to alcohol use by women have increased, largely due, in part, to the increased number of women who consume alcohol regularly. This increase is a paramount concern, as women are more susceptible than men to the effects of alcohol-related liver injury. Health care providers should make efforts to counsel women on the risks of excess alcohol consumption to prevent further increase in alcohol-related liver disease and its associated complications.

## EPIDEMIOLOGY

The prevalence of alcohol use disorder is increasing, and one of the most devastating complications is end-stage liver disease. Interestingly, the consequences of alcohol use do not affect all heavy-drinking individuals with the same frequency. Only 15% of people who drink heavily develop cirrhosis from heavy alcohol consumption.^1^ Certain populations, including those with genetic predispositions (e.g., presence of the *PNPLA3* genotype) and women, are more susceptible to end-stage effects of alcohol-related liver injury.

Historically, alcohol-associated liver injury has been reported to be more prevalent in men, despite women’s increased susceptibility to the detrimental effects of alcohol.^2^ This difference in prevalence largely is due to the fact that men generally consume more alcohol than women. However, a recent study that examined the presence of alcohol-related liver disease from 2009 to 2015 demonstrated increased incidence (50%) of alcohol-related liver injury in women, as compared to a 30% increase among men during the same time period.^3^ The increase in alcohol-related liver injury among women appears to parallel the increase in alcohol consumption observed in women.

A study examining alcohol use patterns in the United States from 2001 to 2002, as compared with 2012 to 2013, reported an 80% increase in heavy alcohol consumption among women and a 30% increase among men.^4^ Similar patterns have been seen globally, with a Japanese study noting a twofold to fourfold increase in alcohol consumption among women from 1968 to 1987.^5^ In this study, the rates of alcohol consumption in men remained static. A meta-analysis examining the effects of alcohol use and cirrhosis reported that cirrhosis was more frequent in women versus men, despite similar amounts of alcohol consumption.^6^

## MECHANISTIC FACTORS

Previous studies have shown that, when controlling for the amount of alcohol consumed and for body weight, women had increased levels of blood alcohol when compared with men.^7^ This increase likely is due to decreased body water content in women, thus leading to a smaller volume of distribution. Moreover, women have reduced gastric alcohol dehydrogenase compared with men and therefore impaired first-pass metabolism, resulting in increased susceptibility to injury.^7^ Additional studies also have shown gender differences in alcohol metabolism by hepatic enzymes such as cytochrome P450 2E1, with lower levels in women due to regulation of growth hormone.^8^ The role of estrogen is also a culprit.

Kupffer cells reside within hepatic sinusoids and play a role in clearance of foreign compounds within the liver. Activation of Kupffer cells leads to cytokine release and subsequent hepatic inflammation.^9^ Rat models have shown that estrogen exposure increases Kupffer cell susceptibility to endotoxin. When animals that received exogenous estrogen were studied, increased Kupffer cell sensitization to lipopolysaccharide was observed.^10^ Additional animal models have demonstrated that increased endotoxin release related to Kupffer cell activation resulted in more severe hepatic injury and necrosis.^11^ In fact, estrogen blockade in mouse models has been shown to attenuate alcohol-related injury in females.^12^

## IMPLICATIONS

These factors likely account for studies showing that women, compared to men, are more susceptible to liver disease with less alcohol consumption, and that women have a faster progression to cirrhosis over a shorter time period. In a study conducted in Australia, the rate of progression to cirrhosis for women was 13.5 years, as compared to 20 years for men, when controlling for less alcohol consumption among the women.^13^ More vexing is that although alcohol abstinence has been linked to fibrosis regression, reports show that among people who had cirrhosis and then abstained from alcohol, women had lower 5-year survival rates than men.^14^

Current recommendations from the “Dietary Guidelines for Americans 2015–2020” advise that women should not consume more than 14 grams of alcohol daily, and men should not consume more than 28 grams of alcohol daily.^15^ The relative risk of alcohol-related liver disease increases in women who drink any more than one drink per day. Recently, the Million Women Study in the United Kingdom published prospective data and reported observed liver disease patterns among women from 1996 to 2001.^16^

An interesting observation from the Million Women Study is that people who reported drinking daily were more susceptible to liver injury than those who reported binge drinking.^16^ Thus, recommendations from this study advise that women abstain from drinking daily. This study also noted that women who drank alcohol with meals were less susceptible to alcohol-related injury than those who drank without eating. A possible explanation for this finding is the increased metabolism of alcohol for those who drank with meals as compared to the metabolism of those who did not drink with meals.

The effects of alcohol consumption outside of meals appear to coincide with the observation that women with eating disorders (e.g., bulimia, anorexia) are more susceptible to alcohol-related liver injury than women with no eating disorder.^17^,^18^ These findings may be explained by the nutritional deficiencies associated with eating disorders, which are hepatotoxic independent of the effects of alcohol. Other studies have shown that increases in alcohol-related liver disease coincide with obesity.^1^ Thus, the presence of eating disorders is not the only risk factor that implicates accelerated progression of alcohol-related liver disease. In a study examining risk factors for liver disease in both men and women, an increased waist-to-hip ratio (a measure of fat distribution) portended a worse prognosis for development of severe liver disease.^1^

## OBESITY AND ALCOHOL USE

A possible explanation for the paradoxical discrepancy between alcohol-related liver injury in people with eating disorders and the recent observed increase in those with obesity may be due to the overlap of non-alcoholic fatty liver disease co-existing with alcohol-related liver disease, thus explaining the latter.

In a non–gender focused study, researchers replaced alcoholic beverages with non-alcoholic beverages to examine the effects on hepatic triglyceride fat content.^19^ Individuals who received a sugary beverage as a substitute for alcohol, as compared with those who received a non-sugary beverage, had increased hepatic triglyceride fat content. Even more intriguing was that the hepatic triglyceride levels for those who consumed the sugary beverage were comparable to the levels observed for those who consumed the alcoholic beverage. The effects of non-alcoholic beverages on the liver warrant further study, but these results may explain the increase of cirrhosis in patients with concomitant alcohol use and obesity.

## MANAGEMENT

Abstinence for individuals with alcohol-related liver injury is paramount to preventing liver-related complications. Although liver disease progression may persist even with abstinence, prevention of further hepatic damage is crucial. After enrolling in alcohol treatment programs, women had higher rates of abstinence than men.^20^ However, women are less likely to use face-to-face counseling and pharmacologic therapy to prevent relapse because of family/childcare barriers and a perceived stigma associated with attending programs.^21^

Moreover, if a woman experiences complications of liver disease and needs a transplant, she is often disadvantaged. A recent study that examined early liver transplantation across multiple centers within the United States reported that few women undergo early liver transplantation for alcoholic hepatitis.^22^ In addition, few women with any type of alcohol-related liver disease receive transplants. In a retrospective study of individuals evaluated for transplantation for alcohol-related liver disease, men were more likely than women to be listed for transplantation.^23^ Also, of all the participants listed, men were more likely than women to receive a transplant.

The lack of proper counseling for alcohol use disorder must be addressed, as studies have demonstrated increased risk of relapse of harmful drinking among women with alcohol-related liver disease who received transplants.^24^ This increased relapse for women is problematic, as it has been associated with a higher incidence of recurrent disease for women than for men.

Determining why women are drinking more and exceeding the drinking observed among men is imperative. Several hypotheses include the paradigm shift of women assuming male gender roles, for example, more women are working outside the home and fewer women are having children.^25^ Another hypothesis is that the increasing stress of family and work balance for women leads to the use of alcohol to manage stress.^26^ In addition, alcohol advertisements targeted toward females have increased, beginning with advertisements for wine coolers in the early 2000s^27^ to the advertisements for “female-friendly” drinks such as wine in the current decade, and have made alcohol use more socially acceptable. Increased alcohol use may inadvertently be used to manage stress.

Research shows that the association between problematic drinking and post-traumatic stress disorder, anxiety, and depression is stronger for women than for men.^28^ Moreover, women are more likely to use alcohol to regulate negative reinforcement, whereas for men, investigators have speculated that drinking results in positive reinforcement.

## FUTURE AREAS OF RESEARCH

It is quite evident from currently available literature that women, compared to men, have an increased risk of end-stage liver disease from alcohol use. Although it has been established that women should consume less alcohol than men, observations vary as to whether binge drinking or moderate daily drinking (i.e., not exceeding 14 grams per day) is more likely to lead to end-stage liver disease. Future studies should be conducted to provide more detailed recommendations, although in the interim, health care practitioners should advise women to consume no more than one drink per day.

In addition, the Million Women Study’s observation that women who did not eat meals while consuming alcohol had increased alcohol-related liver injury needs further corroborative evidence. Currently available literature also indicates that women with obesity should be advised to avoid drinking heavily and to avoid substituting alcohol with beverages that have high sugar content, as these beverages may lead to further hepatic fibrosis despite alcohol abstinence.

Moreover and more significantly, public awareness of current hazardous drinking is needed, as many women are unaware they are increasing their risk of liver disease. Public policies need to minimize alcohol advertising targeted toward women.

## CONCLUSION

Although alcohol-related liver injury previously has not been linked to women, it is paramount to educate women about the dangers of consuming alcohol given that women are more susceptible than men to injury after consuming less alcohol. Globally, alcohol consumption has increased, particularly among women. Safe drinking habits, including not exceeding 14 grams of alcohol consumption in a day, not drinking without eating meals, and avoiding daily drinking, should be recommended. If alcohol use disorder is identified, adequate and appropriate counseling and pharmacologic therapy should be provided. Additionally, further study into the neurobiologic basis leading to alcohol use disorder should be made by clinicians and researchers.
